# Few-Shot Learning in Wi-Fi-Based Indoor Positioning

**DOI:** 10.3390/biomimetics9090551

**Published:** 2024-09-12

**Authors:** Feng Xie, Soi Hoi Lam, Ming Xie, Cheng Wang

**Affiliations:** 1 School of Information Science and Technology, Sanda University, Shanghai 201209, China; c.wang@sandau.edu.cn; 2 Faculty of Science and Technology, University of Macau, Macau 999078, China; soihoi.lam@gmail.com; 3 School of Mechanical and Aerospace Engineering, Nanyang Technological University, Singapore 639798, Singapore; mmxie@ntu.edu.sg

**Keywords:** few-shot learning, indoor positioning, meta-learning, cosine similarity, limited labeled data, few-sample learning

## Abstract

This paper explores the use of few-shot learning in Wi-Fi-based indoor positioning, utilizing convolutional neural networks (CNNs) combined with meta-learning techniques to enhance the accuracy and efficiency of positioning systems. The focus is on addressing the challenge of limited labeled data, a prevalent issue in extensive indoor environments. The study explores various scenarios, comparing the performance of the base CNN and meta-learning models. The meta-learning approach involves few-shot learning tasks, such as three-way N-shot, five-way N-shot, etc., to enhance the model’s ability to generalize from limited data. The experiments were conducted across various scenarios, evaluating the performance of the models with different numbers of samples per class (K) after filtering by cosine similarity (FCS) during both the stages of data preprocessing and meta-learning. The scenarios included both base classes and novel classes, with and without meta-learning. The results indicated that the base CNN model achieved varying accuracy levels depending on the scenario and the number of samples per class retained after FCS. Meta-learning performed acceptably in scenarios with fewer samples, which are the distinct datasets pertaining to novel classes. With 20 samples per class, the base CNN achieved an accuracy of 0.80 during the pre-training stage, while meta-learning (three-way one-shot) achieved an accuracy of 0.78 on a new small dataset with novel classes.

## 1. Introduction

Wi-Fi-based indoor positioning technology has emerged as one of the most widely adopted methods for indoor navigation, primarily due to its extensive coverage and high device penetration. In comparison to other technologies such as Bluetooth, infrared, and video positioning methods, Wi-Fi-based positioning leverages existing wireless network infrastructure, making it suitable for use in environments such as shopping malls, airports, hospitals, and underground parking garages, where it can provide users with navigation assistance, location recommendations, and various other services.

The fingerprint matching method employed in indoor positioning using Wi-Fi signals establishes a mapping relationship between pieces of location information. By directly measuring and acquiring signal characteristics, such as wireless signal strength, this method creates a unique fingerprint corresponding to the positional coordinates of each indoor environment. This fingerprint is then utilized to match the estimated spatial location of the target being measured. The primary advantage of this approach is that it does not require the construction of a channel fading model. However, a notable disadvantage is the complexity involved in creating the fingerprint map, which can compromise data quality due to the effects of wireless signal fading and refraction. Additionally, the labeling and processing of a substantial number of data samples incur significant human and material costs, thereby limiting the large-scale application of indoor positioning technology in practical scenarios.

The application of techniques such as small-sample semi-supervised learning in fingerprint matching has emerged as a significant research focus. Semi-supervised learning leverages unlabeled sample data to supplement a limited quantity of labeled sample data, thereby reducing the reliance on labeled data to some extent. In the context of fingerprint matching, semi-supervised learning algorithms can be employed to extract useful information from unlabeled sample data, enhancing the efficiency of fingerprint database construction. Small-sample learning aims to effectively utilize a restricted number of data samples, and these algorithms can diminish the need for extensive training data while preserving localization accuracy.

This paper investigates the application of few-shot learning in Wi-Fi-based indoor positioning, utilizing Convolutional Neural Networks (CNNs) in conjunction with meta-learning techniques to enhance the accuracy and efficiency of positioning systems. It addresses the challenge of limited labeled data, which is a common issue in large-scale indoor environments. The study compares the performance of base CNN models with that of meta-learning models across various scenarios. The meta-learning approach incorporates few-shot learning tasks such as three-way two-shot and five-way two-shot to improve the model’s ability to generalize from limited data. Experiments were conducted to assess the models’ performance with varying numbers of samples per class (K) after undergoing filtering by cosine similarity (FCS) during both the data preprocessing and meta-learning stages. These scenarios included both base classes and novel classes, with and without meta-learning.

Results indicated that the accuracy of the base CNN model varies depending on the scenario and the number of samples per class retained after FCS. Meta-learning performed well in scenarios with fewer samples. For instance, with 20 samples per class, the base CNN achieved an accuracy of 0.80, while meta-learning reached an accuracy of 0.78. Similar performance trends were observed in other scenarios.

The experiments described in this paper were conducted in a 1200 m^2^ laboratory space on campus, utilizing a dataset consisting of 45 classes (reference points). Future research should aim to enhance dataset diversity to validate the generalizability of FCS and meta-learning across different domains and data types. Additionally, it is essential to investigate the impact of various FCS parameters and their combinations to identify optimal settings for different classification tasks. Further development and testing of advanced meta-learning algorithms are needed to improve performance in few-shot learning scenarios, particularly for tasks with extremely limited data.

## 2. Background

Various technologies can be used for indoor positioning, such as ultra-wideband (UWB) [1], Bluetooth [2], radio frequency identification (RFID) [3], ultrasound [4], and Wi-Fi [5]. Each of these wireless technologies has its own advantages and limitations, and the choice of technology depends on the specific application and environmental factors, as discussed in the review by Obeidat et al. [6].

Wi-Fi positioning technology requires no additional hardware or facilities to be installed, only signal acquisition and processing on top of the existing wireless network. This makes it more cost-effective for large-scale deployment. Wi-Fi-based indoor positioning techniques have been widely studied for their low cost, wide range of applications, and high applicability [7].

The Wi-Fi-based fingerprint matching method for indoor positioning divides the localization area into a number of grid points (localization reference points). Data on signal characteristics such as Wi-Fi radio signal strength (RSS) are collected at each grid point for use as the fingerprint information of the location. The collected data undergo preprocessing, including removal of outliers to ensure data quality. The fingerprint map is constructed through model learning to form the mapping relationship between the location and the signal features.

In the data acquisition and preprocessing stage, multi-dimensional parameter estimation and channel parameters are used to complete the initialization of Wi-Fi signals, and the collected Wi-Fi probe data are parsed, including time, wireless signal strength, and other information, to construct fingerprint map data oriented to indoor locations.

The rapid development and advancement of artificial intelligence has led to increased applications of deep learning. Deep-learning-based models are capable of approximating high-dimensional and highly nonlinear models, therefore enhancing positioning accuracy. Recently, DNN (deep neural network) [8,9], CNN (convolutional neural network), and LSTM (long short-term memory network) [10] models have been introduced to uncover the intrinsic relationships between RSSI data and corresponding positions, achieving better localization performance. Wen Zi Run et al. presented feature extraction of radio signal strength indication (RSSI) data using convolutional neural networks (CNNs) [11]. For example, RSSI is converted to a 2D grayscale image, and features are extracted by depth-separable convolution. A lightweight CNN model can be used to reduce computational complexity and improve positioning accuracy. Experiments show that the method achieves 99% and 99.7% accuracy on the UJIIndoorLoc and Tampere datasets, respectively. There are many appropriate deep learning models that can be used as regressors or classifiers. For example, multilayer perceptron (MLP), recurrent neural network (RNN), and convolutional neural network (CNN) architectures are commonly chosen, as reviewed in the survey of deep learning approaches by Xu Feng et al. [12].

Since the construction of a fingerprint map requires a great deal of labor and material resources, this problem is especially prominent in large-scale indoor environments. In the field of Wi-Fi-based indoor localization, the combination method using few-shot semi-supervised meta-learning has become a research hotspot and is gradually gaining attention. This technique uses a small quantity of labeled data and a large quantity of unlabeled data to improve the generalization ability of the model.

Few-shot learning aims at fast generalization from very small quantities of labeled data. Meta-learning is an effective strategy to achieve fast learning by defining a parameterized model of the learning algorithm and training it on multiple small-sample labeled training sets representing different classification problems [13]. In semi-supervised few-sample learning, a large set of unlabeled data is utilized in addition to a limited quantity of labeled data to further improve the model’s performance.

Noelia Hernández proposed a new system (called WiFiNet) that takes advantage of the strong ability convolutional neural networks in classification problems [14]. The WiFiNet system utilizes the powerful classification capabilities of convolutional neural networks (CNNs) and combines transfer learning and feature extraction methods for indoor localization. Results indicated that, at a medium scale (30 reference positions and 113 access points), it reduced mean error (33%) and processing time compared to state-of-the-art algorithms such as SVM.

In order to solve the problem of insufficient labeled samples in modulation recognition, a smaller-sample recognition algorithm based on pseudo-labeling semi-supervised learning (pseudo-labeling algorithm) was proposed by Shi et al. [15]. In that algorithm, high-quality artificial features, excellent classifiers, and data labeling methods are used to construct an efficient pseudo-labeling system, and then the system is combined with signal classification for deep implementation with either a small number or a large number of unlabeled samples. Simulation results show that the model performance can be improved by 5–10% when the signal-to-noise ratio of six digital signals intended for classification and recognition is greater than 5 dB. 

Wei et al. proposed a strikingly simple approach to semi-supervised few-shot learning (SSFSL) [16]. Similar studies were presented by Wei et al. [17] and Chen et al. [18]. These approaches aim to train a classifier that can adapt to new tasks using limited labeled data and a fixed quantity of unlabeled data. Various sophisticated methods have been proposed to tackle the challenges associated with this problem. Their papers present simple but quite effective approaches to predict accurate negative pseudo-labels from an indirect learning perspective. We leverage this type of augmented support set, which is typically used in few-shot tasks, e.g., one-shot classification. In such label-constrained scenarios, our approach offers highly abundant pseudo-labels. By iteratively excluding them one by one, we ultimately derive positive pseudo-labels for each sample approach. The integration of complements results in significant accuracy improvements for SSFSL. It outperformed state-of-the-art methods on four benchmark datasets. Furthermore, it exhibits good adaptability and generalization capabilities when used as a plug-and-play counterpart alongside existing SSFSL-extended generalized linear models.

Several papers have shown that CNNs have significant advantages in Wi-Fi-based indoor localization [19,20,21]. A novel lightweight CNN model, DS-ECA-CNN, has been designed for multi-building, multi-floor localization scenarios in large-scale indoor environments to improve the localization accuracy and reduce the number of model parameters. The above-cited papers proposed a CNN-based indoor localization model with feature extraction via depth-separable convolution and fixed output using an adaptive pooling layer. In addition, an indoor localization algorithm combining CNN and Wi-Fi fingerprint libraries has also been shown to be effective in improving localization accuracy and reducing computation time.

Small-sample learning was proposed by Li Fei-Fei et al. [22] and Vinyals et al. [23] at a time when, because directly learning a large number of parameters with few samples is very challenging and likely to lead to overfitting, a practical solution was to apply transfer learning. It is possible to train a deep model on a public class (base class) with enough samples and then transfer the model to learn on a new class based on only a few examples. Small-sample learning in the meta-learning framework follows the key idea of learned learning. Specifically, it extracts a small number of learning tasks from the training samples belonging to the base class and optimizes the model to perform well on these tasks. A task typically takes the form of N-way and K-shot, consisting of N classes, each with K support samples (training set) and Q query samples (validation set). (N-way means that there are N classes in the training data, and K-shot means that there are K labeled data points under each class).

Several studies have improved model performance by combining cosine similarity and other metrics. Specifically, one study proposed a negative cosine similarity loss function to enhance stability and avoid overfitting [24]. In addition, there have been studies using cosine similarity to measure the similarity between local feature sets to enhance the generalization of the model [25]. The use of cosine similarity instead of the dot product operation in classification convolutional neural networks has also been explored for effective classification of test feature vectors by Houji Zhou [26].

Few-sample learning methods based on cosine distance and similarity are widely used in computer vision and other fields. These methods enable models to achieve good generalization performance with only a small quantity of labeled data by learning an efficient similarity metric space. Although these methods have made considerable progress, more research is still needed to optimize and improve these algorithms for more complex and diverse application scenarios such as indoor positioning.

Although methods have made some considerable progress in few-sample learning, they still face the following challenges: data representation and distance metrics are key factors affecting the performance of prototype networks. How to represent data more efficiently and select appropriate distance metrics remains an important research topic.

In recent years, semi-supervised meta-learning has become a popular research direction in few-sample learning. The core idea of meta-learning is to train on multiple tasks so that the model can learn a common representation or policy and thus be able to adapt quickly when encountering new tasks. By pre-training classifiers on all base classes and performing meta-learning based on the nearest centroids, significant performance gains have been shown for current state-of-the-art methods [27]. The study proposed a meta-baseline (MB) approach, which outperforms the current state of the art (SOTA) by pre-training classifiers on all base classes and performing meta-learning on a few-shot classification algorithm based on the nearest center of mass.

The important challenge facing few-sample learning is domain variance, i.e., the difference in distribution between base classes and new classes. To address this challenge, several studies have proposed benchmarks for cross-domain few-sample learning and explored different meta-learning and transfer learning approaches to improve the generalization ability of the model. Processing multimodal input data requires more complex network structures and algorithm designs, which increases the computational burden and implementation difficulty of the model.

Studies on few-sample learning have shown that although meta-learning provides an effective framework to improve the generalization ability of models, its effectiveness is affected by a number of factors, including the diversity of tasks, the depth and complexity of the models, and the domain differences between the base class and the new class [28]. Therefore, future research needs to further explore how to design more effective meta-learning frameworks and algorithms to better address these challenges and improve the performance of models in indoor positioning applications.

## 3. Methods

Wi-Fi hotspots/APs (access points) installed in different locations periodically broadcast information such as their MAC address and received radio signal strength (RSS). These data can be collected through end devices (such as mobile phones). The probe data are parsed to extract useful information such as timestamps, BSSID, values (levels) of RSS, reference location ID, etc. One reference location has a set of probe information. They are simultaneously integrated to form a vector, *Loc_i_* = (*RSS*_1_, *RSS*_2_, …, *RSS_n_*), However, Wi-Fi signal data are affected by the complex environment and noise; they are time-consuming and labor-intensive to collect; and they often contain mistakes in data labeling. For error data and for abnormal data detection and processing, it is common to use statistical methods such as mean and standard deviation or machine learning methods such as the isolation forest and support vector machine (SVM) algorithms.

This study proposes a cosine similarity (or cosine distance)-based method for initial preprocessing of raw data. The cosine similarity (abbreviated as “cossim” hereinafter) is achieved by calculating the cosine of the angle between two vectors. The cossim between two vectors, *A* and *B*, is calculated by (*A*·*B*)/*M*(*A*)/*M*(*B*), where (*A*·*B*) specifies the dot product of *A* and *B*, while *M*(*A*) and *M*(*B*) are their second-order Norms, respectively.

In this study, for all raw RSS (Wi-Fi Received Signal Strength) data, every sample is composed of a vector of the form (*RSS*_1_, *RSS*_2_, …, *RSS*_n_), where *n* means the different Wi-Fi devices. These RSS data are grouped into *V_L_* by different reference points L, and the mean values of *V_L_*_,*mean*_ are obtained. The different cosine similarity values between the different pairs of *V_L_* and *V_L_*_,*mean*_ are calculated and sorted in reverse order. The samples with the worst cosine similarity (or largest cosine distance) are removed from the sample (these removed samples are regarded as dirty data or outliers).

**Figure 1:**
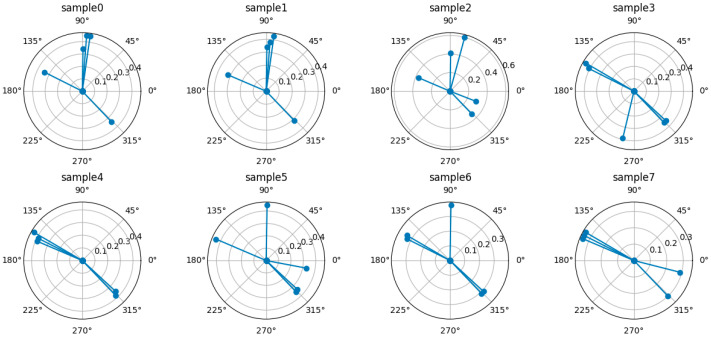
Example of raw sample data (e.g., at a location, ID = 1001).

Figure 1 illustrates and shows 8 raw sample data points. RSS averaging is performed on each sample grouped according to the same reference point, and the five strongest signals are represented as vectors in polar coordinates according to their intensity. The cosine similarity is calculated for all sample vectors, and the sample data with the most similarity at each collection point are retained, while the samples with larger differences (e.g., sample 2) are removed.

In this paper, the fingerprint matching method used for indoor positioning in a real-time environment is treated as a series of optional classification tasks (a universal set of alternatives). For simplicity, the most probable positioning decision points were used as a subset of this universal set. A probability-based measurement is defined as a combination of the features based on probability:

(1) $$U_{ij}=V_{ij}+\varepsilon_{ij}$$

The random utility function *V_ij_* is composed of the features at different reference points. The features includes the different BSSIDs (Basic Service Set IDs, i.e., the MAC addresses of the Wi-Fi devices) and their RSS levels. $\varepsilon_{ij}$ means error, where *i* represents each Wi-Fi device and *j* represents each reference point.

A series of Wi-Fi fingerprint points with the largest random utility function is selected, and their probability values are calculated. In this case, Wi-Fi fingerprint matching is a classification problem.

In standard few-shot classification, given a labeled dataset of base classes with a large number of samples, the goal is to learn concepts in classification with a few samples. In a W-way S-shot few-shot classification task, the support set contains W classes with S samples per class, the query set contains samples from the same W classes with S samples per class, and the goal is to classify the *W* × *S* query into W classes.

**Figure 2:**
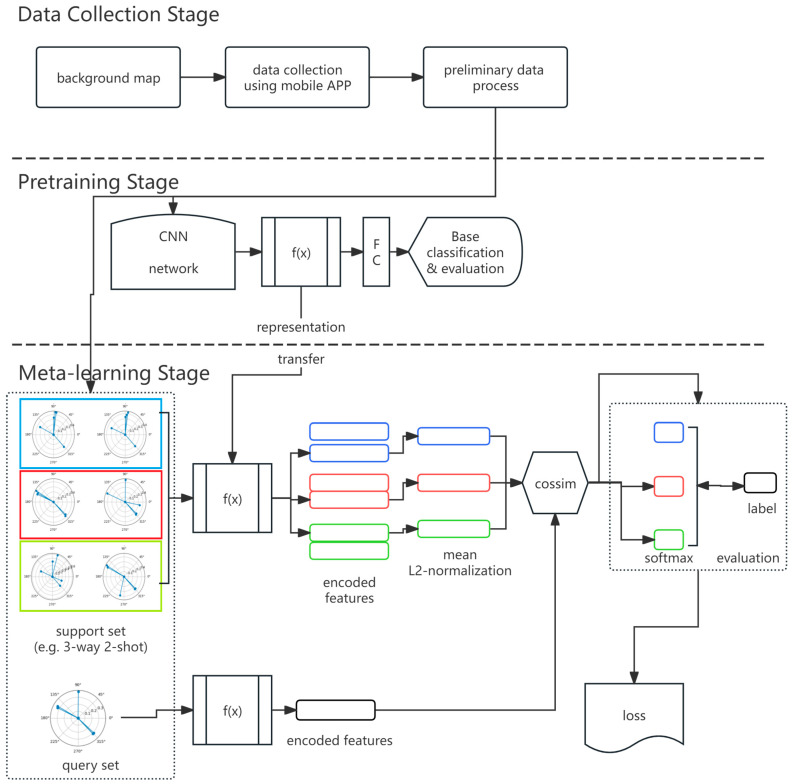
Framework of pre-training and meta-learning.

Figure 2 presents the methodological framework, including the data collection, pre-training, and meta-learning stages.

In Figure 2, f(x) means the function through base CNN layers to obtain the encoded features, and FC specifies the last fully connected (FC) layer. The cossim function calculates the cosine similarity between the encoder vectors of the support set and the query set.

The base classifier is a whole-classification model trained for the whole label set. As pretraining model, it trains a classifier on all base classes with standard cross-entropy loss. In this study, it pretrains a CNN on large-scale training data, then removes its last fully connected (FC) layer and obtains the encoder *f_θ_*, which maps the input to embedding.

The second stage is the meta-learning fine-tuning stage, which optimizes the model on the evaluation metric of the base classifier on the support and query datasets. To compute the loss for each task, it must compute the predicted probability distribution for each sample in query set defined. The loss is the cross-entropy loss computed from the probability *p* and the labels of the samples in the query set. During training, each training batch can contain several tasks, and the average loss is computed.

During this few-shot meta learning, let (*x_i_*,*y_i_*) and *q_i_* be a labeled sample in the support set and the query set, respectively. *f*(*x_i_*) is the feature vector extracted by the pretrained CNN. Fine-tuning is performed by using cosine similarity to compute the logits; it can be helpful to scale the value before applying the softmax function during training (referring to the practices in recent work [29,30,31]).

(2) $$P=Softmax(w\cdot cossim(f(x_{i}),q_{i})+b)$$

where *x_i_* denotes input sample i from the support set and *q_i_* denotes the input sample from query set. The function cossim() means the cosine similarity of two vectors. *w* and *b* are the learnable variables of weight and bias.

## 4. Experiments

The experiment site was located on the fifth floor of a laboratory building (about 1200 m^2^) on campus. The entire modeling and testing took 37 students two full days with a mobile software application (Vips v3.08.2) for collecting Wi-Fi signal data. 

**Figure 3:**
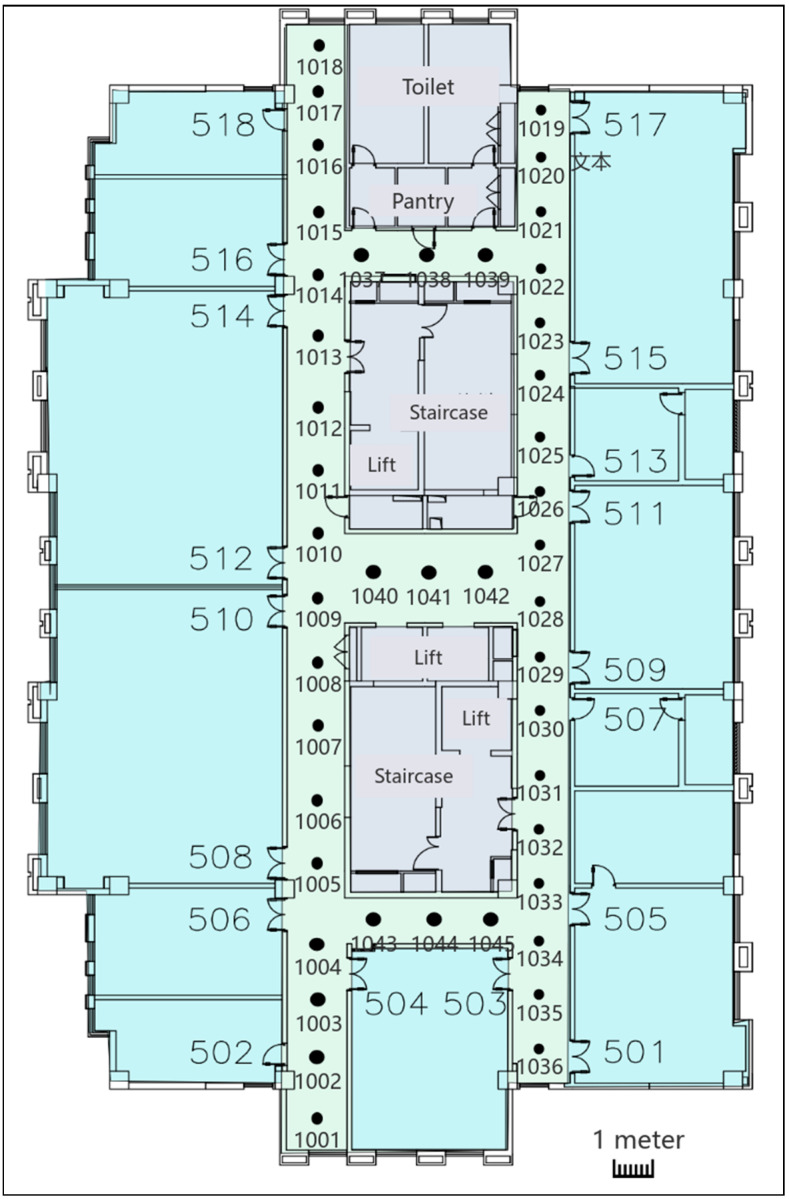
The indoor map for experiments.

The background map (referring to Figure 3) was created using the SVG (Scalable Vector Graphics) image format. In the SVG coordinate system, the *x* and *y* axes represent 2D coordinates (the coordinate origin is in the upper left corner of the map). All geographical features, environmental locations, and coordinate information of the experimental site were pre-recorded.

The research pre-surveyed and recorded the location of each position (coordinate position in the SVG map) and recorded the received signal strength values of multiple Wi-Fi access points at each location. There are 45 reference points, noted as 1001, 1002, …, 1045, located along corridors and in elevator lobbies at about 1–1.5 m intervals. These 45 points were regarded as 45 classifications for further modeling.

Thirty-seven students performed the data collection over several hours. The actual positions on the digital map were recorded manually. Geographical features, such as rooms, doors, and infrastructure around the positioning system, may be used as reference, mapping Wi-Fi fingerprints and their coordinates into a metric system such as a local projected coordinate system. The mobile application was previously deployed for testers collecting data (including raw Wi-Fi data and actual and calculated locations on the map in the app). The software applications installed on the mobile device were less than 50 KB in size, supported by 2.4 G/5.8 G wireless frequencies, and functional across different platforms and manufacturers. The raw data contents included timestamps, MAC addresses, RSS values of the Wi-Fi APs detected, and the IDs and coordinates (*x*,*y*) of reference points. There were 8599 samples collected after removing duplicates. First, a classification model was pre-trained on a large quantity of data (36 of base classes), and then the encoder was retained as a backbone for feature extraction. After pre-training, meta-training is performed on 9 novel classes. The information of samples is given in Table 1.

During the meta-learning stage, the task takes the form of W-way S-shot. For example, for three-way and two-shot, it contains three classes, each with two support samples. The classes (novel classes) in the meta-learning stage are a collection of only a small number of samples and do not intersect with the base classes.

The different models were applied based on the scenarios in Table 2, while the basic CNN model structure is given in Table 3.

For the classification training stage using a basic CNN with the Adam optimizer and a loss function of CategoricalCrossentropy on one GPU, the model ran for 150 epochs with a batch size of 32 and a learning rate of 0.001.

## 5. Results and Discussion

**Figure 4:**
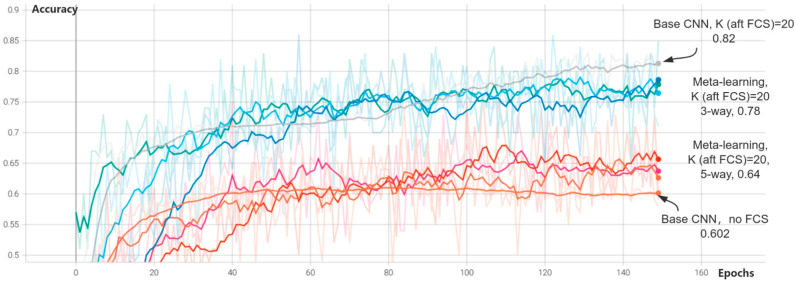
Accuracy of different scenarios.

Scenarios A, B3, C2, and D2 were trained and evaluated according to the defined dataset and models. The average accuracy is shown in Figure 4.

The chart in Figure 4 illustrates the performance of various models in terms of accuracy across epochs. The base CNN model with FCS (K = 20) outperformed the same model without FCS, achieving a higher accuracy of 0.82 compared to 0.602. This finding underscores the effectiveness of FCS in enhancing model performance by retaining the most relevant samples per class. The meta-learning models demonstrated improved accuracy in scenarios with fewer samples per class, particularly when combined with FCS. The three-way meta-learning model with FCS (K = 20) achieved an accuracy of 0.78, which is close to the performance of the base CNN with FCS. Conversely, the five-way meta-learning model exhibits a lower accuracy of 0.64, indicating that the complexity of the meta-learning task influences the model’s performance.

The consistent improvement in accuracy for models utilizing FCS compared to those without it emphasizes the significance of effective data preprocessing in enhancing model performance. Meta-learning using cosine similarity aids in directing the learning process towards the most informative samples, resulting in better generalization and higher accuracy.

The experiment was repeated under various scenarios, with the results summarized in Table 4. This table presents the accuracy outcomes for different scenarios and illustrates the impact of meta-learning on the performance of the base CNN model. The differing experimental settings or conditions are indicated by ‘K after FCS’, which specifies the number of samples per class retained after applying filtering by cosine similarity (FCS).

The accuracy of the base CNN model is reported, with values in parentheses representing macro-averaging, which averages the precision, recall, and F1 scores for each category.

Scenario A serves as the baseline performance without any filtering by cosine similarity (FCS); its accuracy of 0.60 acts as a reference point for assessing the impact of FCS and meta-learning in subsequent scenarios. With 180 samples per class after FCS, the base CNN model demonstrated a slight improvement in accuracy compared to Scenario A. In contrast, reducing the number of samples per class to 20 led to a significant increase in accuracy. The base CNN achieved the highest observed accuracy in the B scenarios with only 20 samples per class. This underscores the effectiveness of FCS in enhancing the model’s accuracy by concentrating on the most relevant features. However, when the number of samples per class was further reduced to 10, a slight decrease in accuracy was observed. This suggests a trade-off between the number of samples and model performance, indicating an optimal range for K after FCS.

The C scenarios involved few-shot learning tasks, with C2 demonstrating the effectiveness of meta-learning by achieving an accuracy of 0.77 with 20 samples per class. This highlights the potential of meta-learning to generalize from limited data. The scenarios D1 represent a five-way two-shot scenario, while D2 achieves an accuracy of 0.65. Similar to the C scenarios, the D scenarios also focus on few-shot learning, with D2 attaining an accuracy of 0.65 with 20 samples per class. This further validates the benefits of meta-learning in enhancing model performance in few-shot settings. Table 4 illustrates the impact of FCS and meta-learning on the accuracy of the base CNN model across various scenarios. FCS significantly improves accuracy by concentrating on the most relevant features, with the optimal number of samples per class being approximately 20. Meta-learning shows promise for further enhancing performance, particularly in few-shot learning scenarios.

## 6. Conclusions and Future Work

This paper investigates the application of few-shot learning in Wi-Fi-based indoor positioning, utilizing convolutional neural networks (CNNs) in conjunction with meta-learning techniques to enhance the accuracy and efficiency of indoor positioning. It addresses the challenge of limited labeled data, which is a common issue in large-scale indoor positioning.

The study demonstrates that data preprocessing through filtering by cosine similarity (FCS) enhance the performance of the base convolutional neural network (CNN) model in classification tasks. The experiments reveal that applying FCS during data preprocessing to reduce the number of samples per class improves the model’s accuracy by concentrating on the most relevant features. The results indicate that FCS effectively enhances the accuracy of the base CNN model by retaining the most pertinent samples per class.

The FCS method is particularly advantageous when the number of samples is significantly reduced, with the highest accuracy achieved using 20 samples per class. However, reducing the number of samples to 10 per class results in a slight decline in accuracy, indicating a trade-off between the number of samples and model performance.

Meta-learning emerges as a powerful approach in few-shot learning scenarios. The meta-learning provides an additional boost in performance in few-shot learning scenarios, underscoring its potential for generalizing from limited data. These findings imply that the integration of FCS and meta-learning can effectively address the challenges posed by limited data in classification tasks, thereby offering a robust solution for enhancing model performance. Meta-learning performed well in scenarios with fewer samples. These samples originate from distinct datasets pertaining to novel classes that were not included in the training of the base CNN on the base classes. For instance, the base CNN achieved an accuracy of 0.80, while meta-learning (for three-way N-shot) reached an accuracy of 0.78. Similar performance trends were observed in other scenarios.

Future research should explore additional areas to build upon the findings of this study. This includes expanding dataset diversity to conduct experiments with a broader range of datasets, thereby validating the generalizability of few-shot classification (FCS) and meta-learning across different domains and data types.

The selected experimental scenario of this study was the environment of a school laboratory building, with almost all Wi-Fi signal sources being fixed. In other complex environments (e.g., shopping malls or indoor airports), multiple additional Wi-Fi sources and mobile sources may appear from time to time. Future studies should investigate the robustness and efficacy of the proposed method and model in the complex scenarios, focusing on practical considerations such as reference point location assignments.

Furthermore, it is essential to investigate the impact of various FCS parameters and their combinations to identify optimal settings for diverse classification tasks. In terms of meta-learning algorithms, developing and testing advanced meta-learning approaches could significantly enhance performance in few-shot learning scenarios, especially for tasks involving extremely limited data. Additionally, considering the integration of FCS and meta-learning with other machine learning techniques, such as transfer learning and reinforcement learning, may lead to the creation of more robust and versatile models.

## Figures and Tables

**Table 1:** Information of samples.

Dataset	Base Classification	Meta-Learning
Series ID of reference points	1001–1036	1037–1045
Number of Wi-Fi APs detected	102	102
Classes	36 (base classes)	9 (novel classes)
Sample size	8599 60% training, 40% validation	3000 (for fine-tuning/testing)

**Table 2:** Information on the scenarios in the experiments.

Scenario Name	FCS ^1^	Num. of Samples per Class (K) ^2^	Class Type (Num. of Classes)	Model (Pretraining, Meta Learning)	Meta-Learning Task (N-Way, Q-Shot)
A	No		Base (36)	Base CNN	
B1 to B4	Yes	(180, 50, 20, 10) ^3^	Base (36)	Base CNN	
C1 to C3	Yes	(50, 20, 10)	Novel (9)	Base CNN + meta learning	(3, 1), (3, 2), (3, 3)
D1 to D3	yes	(50, 20, 10)	Novel (9)	Base CNN + meta learning	(5, 1), (5, 2), (5, 3)

^1^ Whether samples underwent filtering by cosine similarity (FCS) or not. ^2^ Number of the most similar samples retained in each class after FCS, noted as K. ^3^ Experiments were repeated with different K values, respectively, shown in parentheses () for all B, C, and D scenarios.

**Table 3:** Pretraining base CNN structure.

Layer (Type)	Output Shape	Parameters
conv1d (Conv1D)	(None, 100, 32)	128
max_pooling1d (MaxPooling1D)	(None, 50, 32)	0
Dropout	(None, 50, 32)	0
conv1d_1 (Conv1D)	(None, 48, 64)	6208
max_pooling1d_1 (MaxPooling1D)	(None, 24, 64)	0
dropout_1 (Dropout)	(None, 24, 64)	0
Flatten (Flatten)	(None, 1536)	0
Dense (Dense)	(None,102)	156,774
dense_x1 (Dense)	(None, 102)	10,506
l2_normalize (L2_normalize)	(None, 102)	0
dense_1 (Dense)	(None, 36)	3708

**Table 4:** Summaries of accuracy on different scenarios.

Scenario	K after FCS	Base CNN	Meta-Learning		
A	-	0.60 (0.60)			
B1	180	0.64 (0.64)			
B2	50	0.79 (0.79)			
B3	20	0.82 (0.80)		-	
B4	10	0.77 (0.75)			
			Three-way one-shot	Three-way two-shot	Three-way three-shot
C1	50		0.78 (0.78)	0.78 (0.77)	0.79 (0.78)
C2	20	-	0.75 (0.75)	0.77 (0.77)	0.77 (0.77)
C3	10		0.76 (0.76)	0.78 (0.78)	0.79 (0.77)
			Five-way one-shot	Five-way two-shot	Five-way three-shot
D1	50		0.61(0.59)	0.64 (0.63)	0.65 (0.64)
D2	20	-	0.65(0.64)	0.65 (0.64)	0.64 (0.62)
D3	10		0.63(0.61)	0.65 (0.63)	0.63 (0.61)

## Data Availability

The original contributions presented in the study are included in the article/Supplementary Materials, further inquiries can be directed to the corresponding author.

## Supplementary Materials

The following supporting information can be downloaded at: https://www.mdpi.com/article/10.3390/biomimetics9090551/s1.

## References

[1] Alarifi A., Al-Salman A., Alsaleh M., Alnafessah A., Al-Hadhrami S., Al-Ammar M.A., Al-Khalifa H.S. (2016). Ultra Wideband Indoor Positioning Technologies: Analysis and Recent Advances. Sensors.

[2] Spachos P., Plataniotis K.N. (2020). BLE Beacons for Indoor Positioning at an Interactive IoT-Based Smart Museum. IEEE Syst. J..

[3] Magnago V., Palopoli L., Buffi A., Tellini B., Motroni A., Nepa P., Macii D., Fontanelli D. (2020). Ranging-Free UHF-RFID RobotPositioning Through Phase Measurements of Passive Tags. IEEE Trans. Instrum. Meas..

[4] Carotenuto R., Merenda M., Iero D., Della Corte F.G. (2019). An Indoor Ultrasonic System for Autonomous 3-D Positioning. IEEE Trans. Instrum. Meas..

[5] Liu F., Liu J., Yin Y., Wang W., Hu D., Chen P., Niu Q. (2020). Survey on WiFi-based indoor positioning techniques. IET Commun..

[6] Obeidat H., Shuaieb W., Obeidat O., Abd-Alhameed R. (2021). A Review of Indoor Localization Techniques and Wireless Technologies. Wirel. Pers. Commun..

[7] Bellavista-Parent V., Torres-Sospedra J., Pérez-Navarro A. (2022). Comprehensive Analysis of Applied Machine Learning in Indoor Positioning Based on Wi-Fi: An Extended Systematic Review. Sensors.

[8] Shen Z., Zhang T., Tagami A., Jin J. (2021). When RSSI encounters deep learning: An area localization scheme for pervasive sensing systems. J. Netw. Comput. Appl..

[9] Abbas M., Elhamshary M., Rizk H., Torki M., Youssef M. (2019). WiDeep: WiFi-based Accurate and Robust Indoor Localization System using Deep Learning. Proceedings of the 2019 IEEE International Conference on Pervasive Computing and Communications (PerCom).

[10] Jia B., Qiao W., Zong Z., Liu S., Hijji M., Del Ser J., Muhammad K. (2022). A fingerprint-based localization algorithm based on LSTM and data expansion method for sparse samples. Future Gener. Comput. Syst..

[11] Wen Z., Jian X. (2023). A Lightweight CNN-Based WiFi Fingerprint Indoor Positioning Model. Softw. Eng. Appl..

[12] Feng X., Nguyen K.A., Luo Z. (2021). A survey of deep learning approaches for WiFi-based indoor positioning. J. Inf. Telecommun..

[13] Ren M., Triantafillou E., Ravi S., Snell J., Swersky K., Tenenbaum J.B., Larochelle H., Zemel R.S. (2018). Meta-Learning for Semi-Supervised Few-Shot Classification. arXiv.

[14] Hernández N., Parra I., Corrales H., Izquierdo R., Ballardini A.L., Salinas C., García I. (2021). WiFiNet: WiFi-based indoor localisation using CNNs. Expert Syst. Appl..

[15] Shi Y., Xu H., Liu Y. (2020). A Few-Shot Modulation Recognition Method Based on Pseudo-Label Semi-Supervised Learning. Xibei Gongye Daxue Xuebao.

[16] Wei X.S., Xu H.Y., Yang Z., Duan C.L., Peng Y. (2023). Negatives Make A Positive: An Embarrassingly Simple Approach to Semi-Supervised Few-Shot Learning. IEEE Trans. Pattern Anal. Mach. Intell..

[17] Wei X.S., Xu H.Y., Zhang F., Peng Y., Zhou W. (2022). An Embarrassingly Simple Approach to Semi-Supervised Few-Shot Learning. Adv. Neural Inf. Process. Syst..

[18] Chen H., Fan Y., Wang Y., Wang J., Schiele B., Xie X., Savvides M., Raj B. (2022). An Embarrassingly Simple Baseline for Imbalanced Semi-Supervised Learning. arXiv.

[19] Ren Y., Wang X., Liu L., Chen X. (2022). Fast fingerprint localisation based on product quantisation and convolution neural network in a massive MIMO system. Int. J. Sens. Netw..

[20] Qin F., Zuo T., Wang X. (2021). CCpos: WiFi Fingerprint Indoor Positioning System Based on CDAE-CNN. Sensors.

[21] Li S., Huang X. Indoor Fingerprint Positioning Based on WiFi Channel State Information. Proceedings of the 2023 42nd Chinese Control Conference (CCC).

[22] Fei-Fei L., Fergus R., Perona P. (2006). One-shot learning of object categories. IEEE Trans. Pattern Anal. Mach. Intell..

[23] Vinyals O., Blundell C., Lillicrap T., Wierstra D. (2016). Matching networks for one shot learning. Adv. Neural Inf. Process. Syst..

[24] Fu M., Cao Y., Wu J. (2022). Worst Case Matters for Few-Shot Recognition. Computer Vision-ECCV 2022.

[25] Jieyi Y., Yihong D., Jiangbo Q. (2024). Research Progress of Few-Shot Learning Methods Based on Graph Neural Networks. J. Comput. Res. Dev..

[26] Zhou H., Yang L., Bao H., Li J., Li Y., Miao X. (2023). Memristive Cosine-Similarity-Based Few-Shot Learning with Lifelong Memory Adaptation. Adv. Intell. Syst..

[27] Chen Y., Liu Z., Xu H., Darrel T., Wang X. Meta-Baseline: Exploring Simple Meta-Learning for Few-Shot Learning. Proceedings of the 2021 IEEE/CVF International Conference on Computer Vision (ICCV).

[28] Zhan Q., Wang B., Jiang A., Xie X., Zhang M., Liu G. (2024). A two-stage spiking meta-learning method for few-shot classification. Knowl. Based Syst..

[29] Gidaris S., Komodakis N. Dynamic few-shot visual learning without forgetting. Proceedings of the IEEE Conference on Computer Vision and Pattern Recognition.

[30] Qi H., Brown M., Lowe D.G. Low-shot learning with imprinted weights. Proceedings of the IEEE Conference on Computer Vision and Pattern Recognition.

[31] Oreshkin B., Rodríguez López P., Lacoste A. (2018). Tadam: Task dependent adaptive metric for improved few-shot learning. Adv. Neural Inf. Process. Syst..

